# Changes in Health-Related Quality of Life in Patients with Therapy-Resistant Migraine during Treatment with Erenumab in an Ambulatory Care Setting

**DOI:** 10.3390/jcm12175619

**Published:** 2023-08-28

**Authors:** Hannah Haneke, Schirin Sulaiman, Sina Nickel, Bianca Raffaelli, Jan-Peter Jansen, Valerie Kirchberger

**Affiliations:** 1 Heartbeat Medical, 10178 Berlin, Germany; 2 Department of Neurology, Charité Universitätsmedizin Berlin, 10117 Berlin, Germany; 3 Schmerzzentrum Berlin, 10435 Berlin, Germany

**Keywords:** migraine, headache, Erenumab, real-world evidence, patient-reported outcomes, monoclonal antibodies

## Abstract

Migraine preventive treatment with the CGRP-receptor monoclonal antibody Erenumab can positively impact health-related quality of life (HRQoL) and disease-associated disability. Patient-reported outcome measures (PROMs) are a valuable additional datapoint to real-world evidence covering how treatment affects physical, mental, and social domains of patients’ lives. In this real-world, single-center retrospective observational cohort study, we analyzed clinical performance indicators and PROMs for migraine patients who failed at least four other preventive medications and received Erenumab over the course of one year. Endpoints were the average monthly migraine days as well as PROMs including the MIDAS, EQ-5D-VAS and PROMIS-29. Data were collected digitally via the software heartbeat ONE in an ambulatory care setting as part of the clinical routine. A total of 145 patients treated with Erenumab provided data for 12 months. After 12 months, the median number of monthly migraine days decreased from 9 to 7 days. A clinically relevant reduction in migraine days by ≥30% was reported by 40% of the patients. The migraine-specific MIDAS score, the EQ-5D-VAS measuring the overall health status and all PROMIS domains, except sleep disturbance, changed significantly, reflecting a positive disease progression. This study highlights how patients with a treatment-resistant migraine in an outpatient setting benefit from a preventive treatment with Erenumab. A decrease in migraine days and an increase in HRQoL was maintained over one year. It also underscores the significance of collecting real-world evidence, including PROMs, as an integral component of the healthcare cycle, as such data can reveal additional factors relevant to treatment.

## 1. Introduction

Migraine is a chronic and disabling primary headache disorder that is characterized by episodes of throbbing, severe head pain that usually last 4–72 h. Typical associated symptoms are nausea, vomiting and sensitivity to light, sound or movement [1]. Migraine is a complex disorder that significantly decreases patients’ health-related quality of life (HRQoL) in terms of affecting not only physical, but also social and mental aspects of life. Migraine is responsible for a high burden of disease of over 45 million years lost to disability [2].

The use of the CGRP-receptor monoclonal antibody Erenumab has been shown to result in an increase in HRQoL and a decrease in migraine disability [3]. Erenumab is indicated for prophylaxis of migraine in adults who have at least four migraine days per month and was first approved by the FDA in 2018 [4]. At the date of data collection, patients in Germany also had to have a history of at least four unsuccessful previous preventive therapies to be eligible for cost reimbursement by public health insurances [5]. 

Understanding real-world treatment patterns, patient characteristics and safety outcomes for novel treatments is important to practitioners who may be prescribing a new agent with little or no practical experience [6]. Successful management of complex conditions and comorbid conditions can benefit greatly from the use of patient-reported outcome (PROs)-instruments [7]. PROs are data points on results that are assessed directly by the patient, without interpretation by a third party. This is achieved via self-report, generally in the form of digital questionnaires, which serve as instruments for the structured measurement of PROs [8]. These instruments allow us to reveal what matters to patients and track a patient’s change in HRQoL over time, thereby enabling patients to raise issues with clinicians and improving patient–clinician communication and, by means of cohort analysis, allowing us to identify overall trends for certain patient populations [9,10,11]. In line with a holistic health understanding in terms of an integration of physical, mental and social domains, PROs are essential when evaluating treatments and become more recognized as an important additional data point to real-world data analyses [12,13].

The widespread implementation of structured patient-reported outcome measure (PROM) surveys and their evaluation in recent years has been primarily enabled by digital solutions. The reasons for that include scalability and a better user experience for patients, resulting in high response rates of up to 80% [14]. Platforms such as heartbeat ONE, software provided by HRTBT Medical Solutions GmbH (HRTBT) support ongoing real-world data collection with a focus on embedding PRO analysis and interpretation from individual patients in clinical routines. The digital tool aims at a seamless integration of PROs into the day-to-day routine of healthcare providers, thus enabling physicians to stay informed about the health status of their patients and to support better treatment plans. Digital surveillance of Patient-Reported Outcomes (PROs) is progressively being recognized as the standard of care across various medical domains, and it is attributable to numerous benefits for patients in routine clinical practice, including an enhanced physician–patient rapport [15]. 

The objective of this study was to analyze the HRQoL and relevant clinical performance indicators in patients diagnosed with a therapy-resistant migraine and treated with Erenumab. The generation of real-world evidence should especially serve to gain more insights into effective and efficient treatment options in a real-world ambulatory care setting.

## 2. Materials and Methods

### 2.1. Study Design and Procedures

The single-center retrospective observational cohort study was conducted in an ambulatory care setting (Schmerzzentrum Berlin, Schönhauser Allee 172a, 10435 Berlin, Germany). All data were gathered within the context of clinical routine with the primary aim of enhancing the quality of care for individual patients through remote follow-up assessments, initially without the express intent of conducting a study. The retrospective data analysis of clinical routine data of headache patients has been approved by the Charité Ethical Committee (EA1/159/22). No specific ethical approval or written informed consent for participation was required for this study in accordance with the national legislation and the institutional requirements.

Eligible data for this retrospective analysis were obtained from individuals that were (1) ≥18 years and had statutory insurance (2) clinically diagnosed with migraine according to the *International Classification of Headache Disorders 3rd edition* (ICHD-3) (3) prescribed Erenumab and received at least one dose (4) who consented to participate in regular follow-up surveys as part of the regular clinical routine monitoring and consented to data donation via a general consent form. A total of 184 patients received at least one Erenumab prescription within the inclusion period. They were recruited, treated and supervised by doctors at a specialized ambulatory care center. 

The average monthly migraine days were evaluated at least once every 3 months at visits to the care center as part of the clinical anamnesis. Specifically, patients were asked how many average migraine days they experienced in the past 30 days. These data were manually entered into the software for further analysis. Compared to the baseline, a reduction of at least 30% or 50% in the number of migraine days is considered clinically relevant.

Additionally, further anamnesis questions, clinician-reported outcomes (CROs, such as headache days) and PROs as well as care measures (time and frequency of application, changes in dosing, discontinuation) and level of patient support intensity (physical and remote (mail, phone) consultations, none) were recorded at admission and at predefined follow-up times after initial treatment at 1, 2, 3, 6, 9 and 12 months, which correspond to T0-T6. The surveys were completed on-site via a tablet or remotely via email. PRO instruments included the Migraine Disability Assessment (MIDAS), the PROMIS-29 as well as the EQ-5D-VAS.

#### 2.1.1. MIDAS

The MIDAS is a brief, self-administered, seven-item questionnaire that was developed for the assessment of headache-related disability in three domains: employment (including schoolwork or paid work), household work and non-work activities (including family, social or leisure activities). Patients are asked to indicate the number of days missed due to their headache for all domains over the past three months (Question 1, 2 and 5). Additionally, they are asked to record the number of additional days with significant limitations to activity (defined as ≥50% reduced productivity) in the domains of employment and household work for the same time period (Question 2 and 4). The overall MIDAS score is calculated as the sum of responses for Questions 1 to 5. Two further questions assess the frequency of headaches and pain intensity. They are not taken into account for the MIDAS score but rather serve to provide clinically relevant information to the doctor. Four grades of severity were defined: Grade I (minimal or infrequent disability) with a score of 0–5, Grade II (mild or infrequent disability) with a score of 6–10, Grade III (moderate disability) with a score of 11–20 and Grade IV (severe disability) with a score of 21 or more [16]. The MIDAS has been shown to be a valid and reliable assessment tool for migraine care. A translation into the German language has also been validated and used for this study [17].

#### 2.1.2. PROMIS

The PROMIS-29 measures HRQoL with a fixed number of 29 items from seven core domains: pain (intensity and interference), fatigue, depression, anxiety, sleep disturbance, physical function, and satisfaction with participation in social roles. Each of the seven domains is assessed with four questions and answers are rated on a 5-point Likert scale. Additionally, pain intensity is assessed as a numeric rating item from 0 to 10. For the scoring of PROMIS measures, the T-score metric applies (*M* = 50, *SD* = 10) and high scores represent a high manifestation on the construct being measured. Thus, a score of 50 represents the average of the reference population and a score of 60 would allocate the person one standard deviation above the reference population [12,18,19,20].

#### 2.1.3. EQ-5D-VAS

The EQ-5D-VAS is part of the EQ-5D questionnaire, which is a generic self-report instrument for the assessment of overall health status. Patients are asked to rate their overall health for the day of the assessment on a visual analogue scale from 0–100. A higher score indicates better health [21].

### 2.2. Data Collection

Data were collected via heartbeat ONE, a web-based medical device software used to gather, visualize and export structured CRO- and PRO-data. The platform is provided with usage costs by HRTBT Medical Solutions GmbH (HRTBT) to support ongoing real-world data collection with a focus on embedding PRO analysis and interpretation from individual patients into clinical routines. The system sent questionnaires automatically at the corresponding time point, as well as up to three reminder notifications if a patient was not responding or had not completed the survey yet. While heartbeat ONE was provided by HRTBT, the data were collected solely by Schmerzzentrum Berlin (SZB) via the platform. The data were stored at a GDPR-compliant data center in Germany. All patients included in this report consented to their data being used for possible retrospective analysis, as conducted in this study. Their records were de-identified prior to study cohort selection and were stored encrypted in said data center.

### 2.3. Analysis

Descriptive statistics were performed for the demographic and clinical data, derived from the anamnesis using R (version 4.0.5) package tableone (version 0.13.0) and Excel. In the next step, we analyzed and established a simple real-world evidence marker widely used to evaluate the effectiveness and efficiency of migraine medication: the amount of migraine days in the last month. The Friedman rank test and Cohen’s d were used to determine the influence on migraine days over follow-up timepoints grouped by patient ID, including only patients with a complete data set. The Wilcoxon rank test was used to identify significant multiple pairwise comparisons between the timepoints. The reduction in migraine days from baseline was calculated across the different follow-ups in order to determine clinical relevance. Clinically relevant patient data were counted when a reduction of 50% was shown. In addition, the package ordinal (version 2019.12.10) was used for a cumulative link mixed model with the endpoint “MIDAS score”. Time was defined as a predictor of the models, with T0 (baseline) as the reference. The models were controlled for sex, BMI, and duration of drug use, while the patient ID was defined as the random effect.

HRQoL was analyzed through data of PROM-Instruments of the study cohort. Test statistics performed were one-way ANOVA on continuous scores and Chi-squared test on categorical scores. A value of *p* < 0.05 was considered statistically significant. There was no imputation for missing values and all patients with missing values were excluded from the prediction model. A correlation test was computed using the R package tidyverse (version 1.3.1) and Spearman method for nonparametric measures between all patient-reported outcomes and the clinically established parameter of “Migraine days in the last month”.

## 3. Results

### 3.1. Descriptive Statistics on Demographic and Clinical Characteristics of the Study Population

In total, 145 (78.8%) out of 184 patients treated with Erenumab received therapy for 12 months and were included in the descriptive statistics. Of these, 122 identified as female (84.1%) and 23 as male (15.8%). A vast majority (114 patients (78.6%)) were diagnosed with a chronic migraine and 31 patients (21.4%) were diagnosed with an episodic migraine. The median age at initiation of Erenumab (IQR) was 52 years, with a median BMI of 24.5 kg/m^2^. Further, 104 patients (71.7%) were in a relationship and 95 (65.6%) were employed either full or part time. Sixteen patients (11%) were unable to work due to their migraines. The most frequent comorbidities included depression (24.1%), high blood pressure (20%) and joint diseases such as arthrosis (17.2%) (Table 1).

### 3.2. Characteristics of Treatment

The majority of patients (97%) began treatment with an Erenumab dosage of 70 mg. However, around 30% experienced a dosage adjustment in terms of an increase to 140 mg, which predominantly occurred in the first month after initiation according to the established protocol at the care center (Table 2a). One-fifth of the study population (n = 39, 21,2%) interrupted the treatment, in most cases due to a lack of efficacy. Side effects or planned therapy pauses occurred less frequently (Table 2b).

### 3.3. Monthly Migraine Days

**Figure 1:**
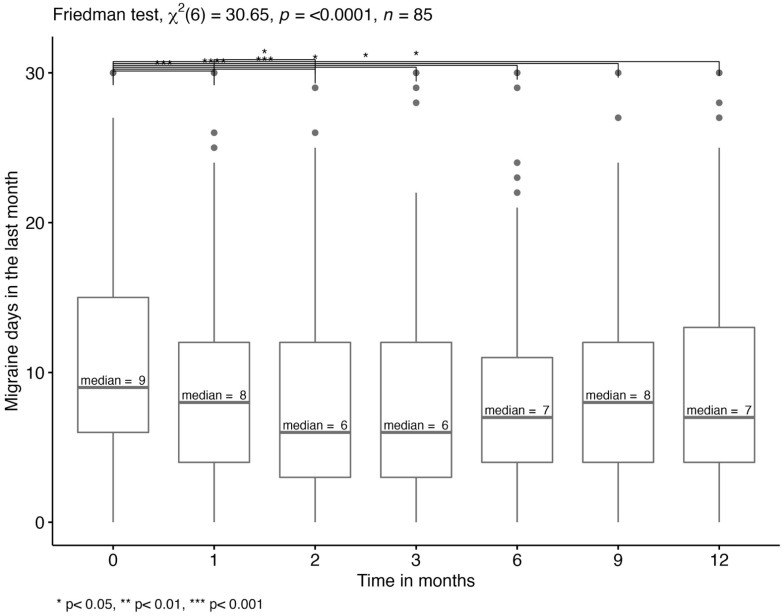
Monthly migraine days. Multiple pairwise-comparison for MMD in the last month using Wilcoxon rank test between different timepoints. The median migraine days are displayed.

The median number of migraine days in the last month (monthly migraine days, MMD) over different timepoints were analyzed for patients with a complete data set regarding this variable (n = 85). According to the Wilcoxon rank test, there were significant differences in MMD between T0 and T1 to T6 and between T1 and T2 (*p* < 0.05). Thus, the MMD (IQR) significantly decreased from 9 (6, 15) to 6 (3, 12) within the first two months after treatment initiation and to 7 after one year of treatment (Figure 1).

A ≥30% reduction in migraine days was reported by 40% and a ≥50% reduction by 31% (≥50%) of patients after 12 months (Table 3).

### 3.4. Patient-Reported Outcomes: MIDAS Score

**Figure 2:**
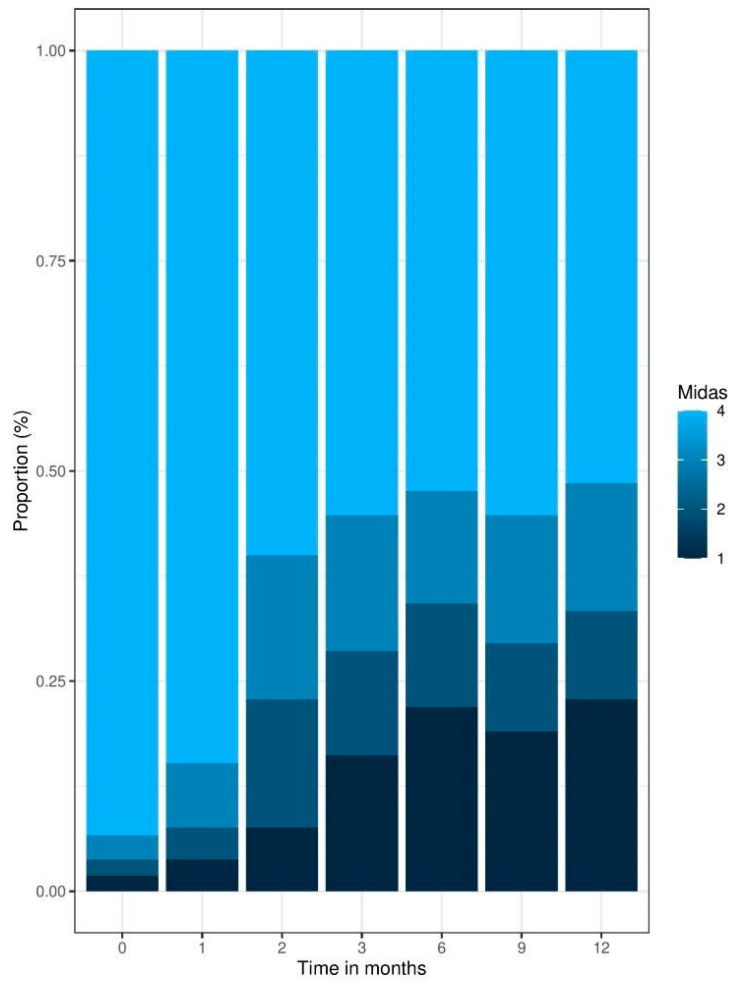
Patient-reported outcomes: MIDAS Score Time Series proportion barplot.

Regarding headache-related disability, a proportion shift of the MIDAS severity grades from 4 (Grade IV, severe disability) to 1 (Grade I, minimal or infrequent disability) can be observed for different time points (Figure 2). In total, 93% of patients showed a MIDAS severity grade of 4 at the beginning of their treatment. By the end of the 12-month period, the amount showing grade 4 severity was reduced to 51%. This effect was found to be significant by performing a chi-square test of independence, *X*^2^ (18, *N* = 85) = 94, *p* < 0.001.

### 3.5. Patient-Reported Outcomes: EQ-5D-VAS and PROMIS

The median of the EQ5D-VAS score measuring overall health status increased significantly over time (*p* < 0.01). It reached an increase of +13/100 score points after 12 months from an initial score of 56 to 69, with the greatest change detected in values before and directly after initiation of Erenumab.

Regarding the PROMIS domains of anxiety, depression, pain intensity and pain interference, lower values indicate better health, whereas higher values in the domains of physical function and social role/activity indicate a better quality of life. All of the domains significantly change over time, except for sleep disturbance (*p* = 0.5). Each of them increases or decreases, indicating an improvement in the respective domain (Table 4).

### 3.6. Correlation Analyses

**Figure 3:**
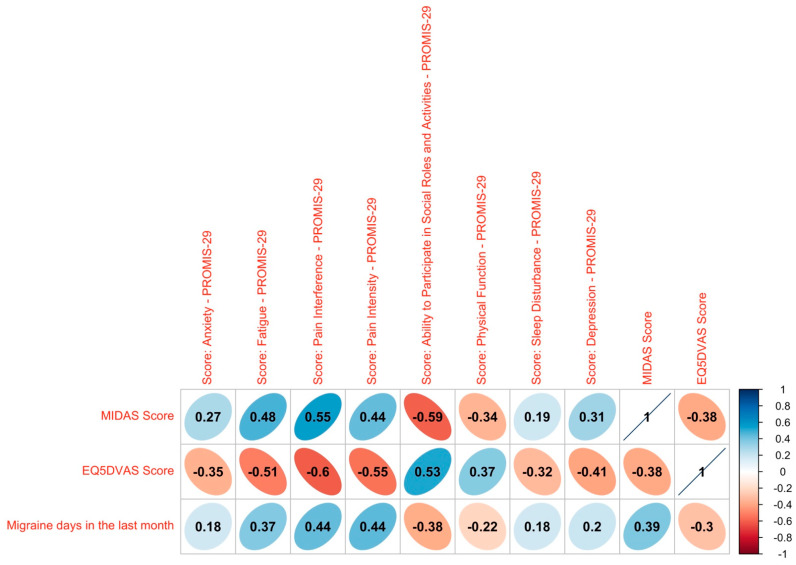
Correlation analysis. Spearman correlation between MIDAS score, EQ5D-VAS score and PROMIS domains with the migraine days. Significant values are plotted. Rank correlation coefficients: ≤0.05: no correlation, <0.05 to ≤0.20: weak, >0.20 to ≤0.50: medium strong, >0.50 to ≤0.70: strong, >0.70: very strong.

To visualize the relationship between the patient-reported outcomes themselves and the clinical marker of migraine days in the last month, a Spearman correlation was calculated (Figure 3). The MIDAS score shows a weak positive correlation with the migraine days in the last month and the EQ-5D-VAS shows a medium strong negative correlation, indicating that they both display the same direction but that the use of these two instruments might generate added value. It becomes evident that all of the PROMIS correlations plotted are significant with medium strong correlations for most domains and low correlations for sleep disturbance, depression and anxiety. The ability to participate in social roles and activities as well as the physical function are negatively correlated with the migraine days, whereas the domains of pain interference and intensity, sleep disturbance, fatigue, depression and fear are positively correlated with the headache days. In general, a higher quality of life is associated with fewer headache days, indicating that the PROMIS score, the EQ-5D-VAS and the MIDAS are good indicators for the overall health of the patients.

## 4. Discussion

This study shows how patients with a therapy-resistant migraine can profit from receiving a preventive treatment with the monoclonal antibody Erenumab. Evidence supporting this finding includes a significant decrease in the number of migraine days, as well as an improvement in outcomes measured by MIDAS, EQ5D-VAS and PROMIS-29 domains, which reflects an increase in HrQoL. Furthermore, the study demonstrated how digital monitoring with PROMs as part of real-world, routine data collection can generate new findings on best treatment options in an ambulatory care setting.

The observed significant decrease in migraine days during the first few months is in line with previous results reported by Scheffler et al. for a similar therapy-refractory population in which the number of MMD decreased by ≥50% after three months for 57.7% of episodic migraine patients (−3.43 days) and 41.9% of chronic migraine patients (−4.72 days) [22]. Lower results were reported by Raffaelli et al., with around 30% of therapy-resistant chronic migraine patients achieving ≥50% response after 3 months. The MMD significantly reduced by 4.5 days from 15.4 to 10.9 days after 9–12 weeks of treatment [23]. However, it has to be taken into consideration that patients’ self-reporting of monthly migraine days at 3 months visits might be subject to a reporting bias.

Similar to our finding of an unsteady development of number of migraine days beyond three months, de Vries Lentsch et al. reported a ≥50% (≥30%) reduction in MMD for 36% (60%) of patients in at least 3 out of 6 months, but only 6% (24%) had this steady reduction in all 6 months [24]. It should be noted that 13.5% of the patient cohort discontinued Erenumab usage, due to insufficient efficacy. Future research endeavors are encouraged to explore the disparate effectiveness observed among patients and delve into the underlying causes of this phenomenon.

Also, in a recent study by Ashina et al., an improvement considered as clinically meaningful in episodic migraine patients was only achieved beyond 12 months; thus, it would be interesting to extend the observation period [25].

The real-world evidence for other antibodies targeting the CGRP pathway, including Fremanezumab and Galcanezumab, is limited. While the existing studies suggest a greater reduction in MMD with these CGRP ligand antibodies, population differences make a direct comparison with our results impossible [26,27]. Most importantly, a high number of prior preventive treatments is a known predictor of poor treatment response, which might explain the lower response rates in this treatment-refractory population [28].

Nevertheless, a significant number of patients reported a clinically meaningful improvement in migraine days of 50%, respectively, 30%. While in preventive episodic migraine treatment, a 50% reduction in migraine days is considered to be clinically relevant [29], it was proposed to lower the cut-off value to 30% for patients with chronic migraines that are more difficult to treat [30] and there are even ongoing attempts to classify an MMD reduction of ≥30% for all migraine patients as clinically relevant [24]. Especially for a population that is considered hard to treat, with no other therapeutic options and highly frequent attacks, this can be a meaningful improvement since patients who were failing more migraine prophylactics have shown a lower response rate before [31]. Practically speaking, decreasing migraine frequency even by a few days can reduce economic and social impacts that result from impairments in normal day-to-day tasks or productive work [25].

Psychiatric comorbidities are especially common in patients with a treatment-resistant migraine, a finding confirmed by the most-commonly associated comorbidities in our study [32]. Relevant comorbidities, e.g., fibromyalgia, should be investigated in more detail. This further stresses the relevance of assessing other health aspects as was the case in this investigation, since high rates of depression in particular can be one of the reasons for chronification [33]. High depression comorbidity could also have affected compliance to treatment and thus impact dropout rates.

A positive correlation was observed between MMD and depression, anxiety, and sleep disturbance; however, the association was weaker than initially expected. Despite this, the correlation remains statistically significant, and enhancing the test power could potentially unveil a more robust relationship. It is crucial to acknowledge that our sample excluded individuals with severe depression or related conditions, which may have influenced the correlation’s strength.

Capturing HRQoL via patient-reported outcomes has been rated by physicians as an important aspect in addition to clinical outcomes such as the amount of migraine days [34]. Regarding headache-related disability measured using the MIDAS, treatment is rated as effective when a >30% reduction after three months is achieved [35], which can be observed in the present results. In a previous study by Lipton et al. (2019), 81.9% of participants had MIDAS scores of ≥21 (Grade IV, severe disability) and 61.4% had scores of ≥41 (very severe disability) which improved after three months by −19.4 days (70 mg) and −19.8 days (140 mg) [36]. The present study fills an important gap by extending the assessment of headache-related disability to a longer follow-up period of 12 months, demonstrating a reduction in patients reporting the worst severity grade of 4 from 93% (T0) to 51% (T12).

Overall health status measured by the EQ5D-VAS score increased significantly over time, indicating that the treatment can be successful long term to help patients enjoy more migraine-free days as well as better health. Patients also tended to present with less anxiety, depression, fatigue, pain interference and intensity and showed an improvement in social role/activity as well as physical function one year after treatment initiation (measured by PROMIS domains).

To our knowledge, this study is the first to assess the broad collection of PROMIS domains regarding migraine treatment with Erenumab. Only pain interference has been assessed with these instruments by Lipton, including patients with chronic migraine [36]. Furthermore, no minimally important differences have been defined for the PROMIS instruments in a migraine cohort or other pain patient populations, making it difficult to fully interpret score changes as clinically meaningful. Only for pain intensity, the Institute for Quality and Efficiency in Health Care (IQWiG) suggests a threshold of 15% compared with baseline as a clinically relevant [37]. An integration of these domains into clinical trials in order to assess relevant aspects regarding HRQoL might be useful as part of a scientific routine.

The correlation analyses between the PROMs themselves, as well as the clinically established marker of migraine days in the last month, all point in the right direction and show the relevance of assessing the patients’ perspective. However, bearing in mind that the correlations were partly weak, the collection of PROMs might add a significant value to the collection of established clinical markers, which is in line with previous studies [10].

We found relatively low dropout rates of ⅕, similar to the results of Robblee et al. (2020) who reported that 27.7% of patients discontinued treatment primarily due to adverse events or ineffective treatment [38]. In the present study, most dropout patients terminated the treatment due to a lack of efficacy and only a small amount due to adverse effects, underlining the good tolerability of Erenumab.

### Limitations

This study also presents several limitations. Since 84% of the study population was female, it is important to consider the limited possibility of drawing conclusions regarding the male population. The female share in our study was higher than compared to two German database studies [39,40], which might be explained by having included a comparably sicker and treatment-resistant patient population. In similar studies including this or an even sicker population that had tried more preventive medication, the number of women was equally high [24] or even higher [38]. Further, the study was conducted with a very heterogeneous study population regarding the type of migraine or comorbidities. The number of patients with CM has been greater in other investigations [38] and they report significantly higher comorbidities, which can have an important impact on HRQoL [41]. Due to the retrospective design of the study, further information on patients’ clinical history besides receiving four prior medications was not collected in detail. For a full interpretation of the results, this should be addressed thoroughly. Also, former studies have shown a great number of patients (>50%) returning from chronic to episodic migraine after treatment [22]. A subgroup analysis for episodic and chronic migraine patients on different follow-up timepoints should be considered in future studies.

An additional constraint encountered in this investigation pertains to its retrospective nature. Given that real-world evidence (RWE) data were collected in a routine care setting, the absence of a control group became evident, thereby limiting the statistical scope to longitudinal assessments exclusively. Consequently, we advocate for the design of future investigations as prospective study designs, which should incorporate RWE data supplemented with randomized controlled trial (RCT) data. Critical to this proposition is the inclusion of a control group, which would significantly enhance the robustness and validity of our research findings. Furthermore, we recommend increasing the sample size and extending the observation period beyond 12 months. Regarding the outcomes, the number of MMD were based on self-reports rather than headache diaries, which could have caused uncertainties about the validity of such an outcome measure. Also, most studies on preventive migraine treatment collected different patient-reported outcomes compared to the study at hand.

## 5. Conclusions

More insights on patients treated in ambulatory care settings not only shed light on treatment itself but also the environment and its influence. Treatment with Erenumab decreased MMD after 12 months and also the measured PROs showed a significant improvement in the patients’ quality of life in relation to the therapy, resulting in increased social and physical activity. Moreover, these improvements in quality of life were not only achieved shortly after the initial dose, but were also maintained during the later observation period, with decreasing care and support efforts. Further research including outcome measurement can provide a deeper understanding of patients’ health and the effectiveness of the treatment.

## Figures and Tables

**Table 1:** Descriptive statistics on demographic and clinical characteristics of the study population.

Characteristic	*N* = 145 ^1^
Age	52 (45, 58)
Female	84.1 (122)
BMI	24.5 (21.5, 27.3)
Type of migraine	
Chronic	78.6 (114)
Episodic	21.4 (31)
Relationship status	
Married/in a relationship	71.7 (104)
Unmarried/single	23.4 (34)
Divorced/separated	4.1 (6)
Widowed	0.7 (1)
Work status	
Working full-time	42.8 (62)
Working part-time	22.8 (33)
Not working by choice	13.1 (19)
Unable to work due to migraine	11.0 (16)
Unable to work due to another condition	8.3 (12)
Seeking employment	2.0 (2)
Comorbidities	
None	37.2 (54)
Depression	24.1 (35)
High blood pressure	20.0 (29)
Joint diseases	17.2 (25)
Autoimmune diseases	9.0 (13)
Gastrointestinal diseases	9.7 (14)
Anxiety disorder	8.3 (12)
Cancer	4.1 (6)
Anemia	4.1 (6)
Kidney diseases	2.8 (4)
Liver diseases	2.1 (3)
Diabetes	2.1 (3)
Heart diseases	2.1 (3)

^1^ Median (IQR); % (*n*).

**Table 2:** Characteristics of treatment. (**a**) Interruption of treatment. (**b**) Dose proportion over time.

(a)							
Time in months	0, *N* = 145 ^1^	1, *N* = 145 ^1^	2, *N* = 145 ^1^	3, *N* = 145 ^1^	6, *N* = 145 ^1^	9, *N* = 145 ^1^	12, *N* = 145 ^1^
Dose							
70 mg	140 (97%)	114 (79%)	105 (72%)	100 (69%)	99 (68%)	96 (66%)	96 (66%)
140 mg	5 (3.4%)	31 (21%)	40 (28%)	45 (31%)	46 (32%)	49 (34%)	49 (34%)
**(b)**							
**Characteristic**					***N* = 39 ^1^**		
Reason for interruption of treatment							
Lack of efficacy					25 (64%)		
Planned therapy break					4 (10%)		
Side effects					5 (13%)		
Other					5 (13%)		

^1^ *n* (%).

**Table 3:** Clinically relevant reduction in migraine days. Patient proportion with a ≥30% and ≥50% reduction in migraine days in comparison to baseline (clinical relevance).

Time in Months	1, *N* = 85 ^1^	2, *N* = 85 ^1^	3, *N* = 85 ^1^	6, *N* = 85 ^1^	9, *N* = 85 ^1^	12, *N* = 85 ^1^
Clinical Relevance ≥ 30%	27 (32%)	41 (48%)	40 (47%)	36 (42%)	38 (45%)	34 (40%)
Clinical Relevance ≥ 50%	17 (20%)	28 (33%)	30 (35%)	26 (31%)	26 (31%)	26 (31%)

^1^*n* (%).

**Table 4:** Patient-reported outcomes: EQ-5D-VAS and PROMIS test statistic using one-way ANOVA on PROM scores over time in months.

Characteristic	0, *N* = 105 ^1^	1, *N* = 105 ^1^	2, *N* = 105 ^1^	3, *N* = 105 ^1^	6, *N* = 105 ^1^	9, *N* = 105 ^1^	12, *N* = 105 ^1^	Test Statistic	*p*-Value ^2^
EQ5DVAS Score	56.0 (45.0, 75.0)	70.0 (54.0, 80.0)	70.0 (53.0, 81.0)	74.0 (57.0, 85.0)	70.0 (50.0, 80.0)	70.0 (50.0, 84.0)	69.0 (51.0, 81.0)	3.3	0.003
Score: Anxiety—PROMIS- 29	55.8 (48.0, 59.5)	53.7 (48.0, 57.7)	51.2 (48.0, 55.8)	48.0 (40.3, 55.8)	51.2 (40.3, 55.8)	51.2 (40.3, 57.7)	51.2 (40.3, 57.7)	4.4	< 0.001
Score: Fatigue—PROMIS- 29	57.0 (51.0, 64.6)	53.1 (48.6, 60.7)	53.1 (48.6, 58.8)	51.0 (48.6, 58.8)	51.0 (48.6, 60.7)	51.0 (48.6, 60.7)	53.1 (48.6, 58.8)	3.5	0.002
Score: Ability to Participate in Social Roles and Activities—PROMIS-29	42.3 (37.3, 48.1)	44.2 (42.3, 51.9)	44.2 (42.3, 51.9)	48.1 (42.3, 51.9)	48.1 (42.3, 51.9)	46.2 (42.3, 51.9)	46.2 (42.3, 51.9)	4.7	< 0.001
Score: Physical Function— PROMIS-29	41.8 (36.7, 45.3)	41.8 (37.9, 45.3)	43.4 (40.4, 56.9)	45.3 (39.1, 56.9)	45.3 (40.4, 56.9)	45.3 (40.4, 56.9)	45.3 (40.4, 56.9)	5.3	< 0.001
Score: Sleep Disturbance— PROMIS-29	52.4 (46.2, 57.9)	52.4 (46.2, 56.1)	50.5 (43.8, 57.9)	50.5 (46.2, 54.3)	50.5 (43.8, 56.1)	52.4 (43.8, 57.9)	50.5 (46.2, 57.9)	0.90	0.5
Score: Depression— PROMIS-29	53.9 (49.0, 58.9)	51.8 (49.0, 57.3)	51.8 (41.0, 57.3)	51.8 (49.0, 57.3)	51.8 (49.0, 55.7)	51.8 (49.0, 57.3)	53.9 (49.0, 57.3)	2.5	0.022
Score: Pain Interference— PROMIS-29	62.5 (59.9, 66.6)	57.1 (53.9, 62.5)	57.1 (53.9, 62.5)	57.1 (53.9, 61.2)	55.6 (53.9, 62.5)	58.5 (53.9, 62.5)	57.1 (53.9, 62.5)	6.0	< 0.001
Score: Pain Intensity— PROMIS-29	7.0 (5.0, 7.0)	4.0 (2.0, 7.0)	4.0 (3.0, 6.0)	4.0 (3.0, 6.0)	4.0 (2.0, 6.0)	5.0 (2.0, 6.0)	5.0 (2.0, 7.0)	5.8	< 0.001

^1^ Median (IQR). ^2^ One-way ANOVA.

## Data Availability

The data presented in this study are available on reasonable request from the corresponding author.
